# Supplementary material for: Transiently antigen-primed B cells return to naive-like state in absence of T-cell help
Source: Nat Commun. 2017 Apr 21;8:15072. doi: 10.1038/ncomms15072 (PMC5413946; doi:10.1038/ncomms15072)
Supplement: Supplementary Information — Supplementary Figures and Supplementary Table [file ncomms15072-s1.pdf]

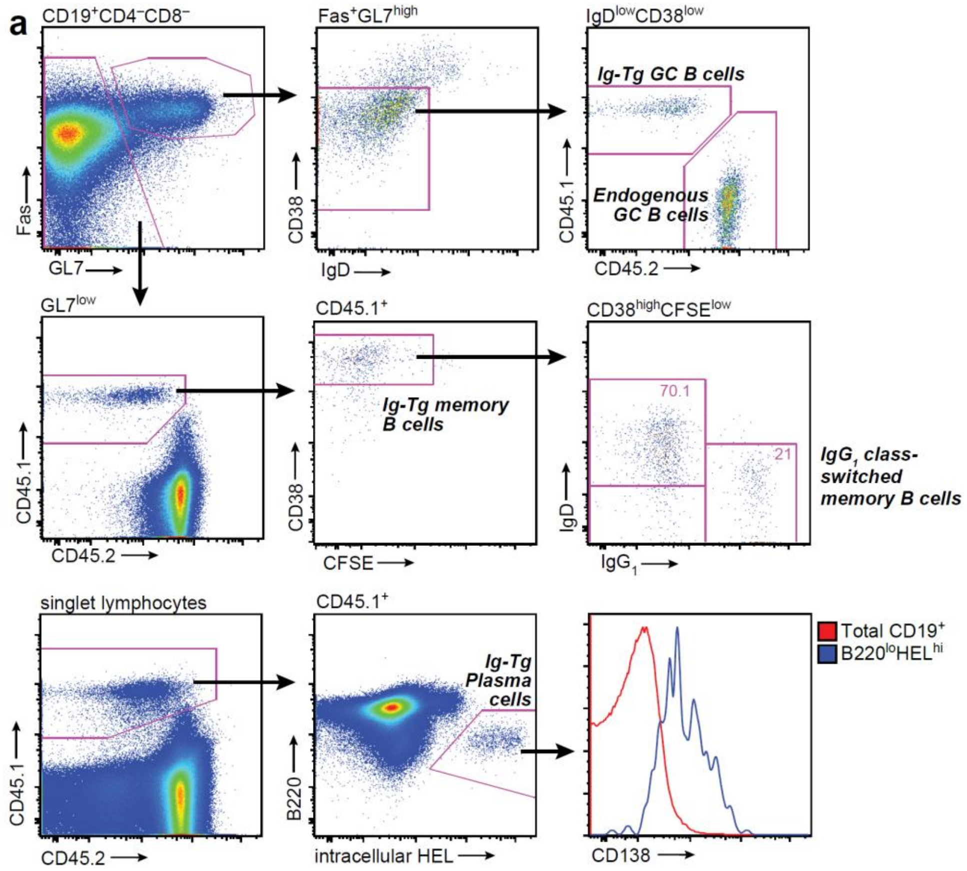


**Supplementary Figure 1 (relates to Fig. 1)**. **A single exposure to Ag enables B cell participation in the GC, memory B cell, and PC responses *in vivo*. a**, Ig-Tg HyHEL10 GC, memory B cell, $IgG_1$ class-switched memory B cell, and PC gating strategies.

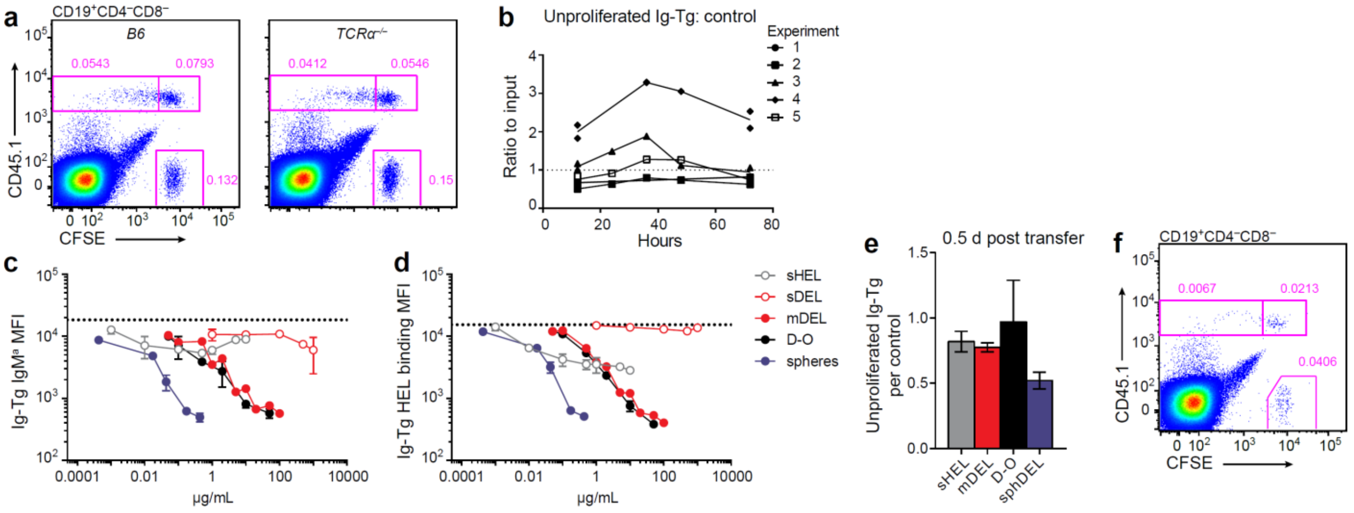


**Supplementary Figure 2 (relates to Fig. 3)**. **Ag-primed B cells do not undergo apoptosis in the absence of T cell help *in vivo*. a**, **b**, Time-course analysis of DEL-OVA-pulsed (50 μg/mL) Ig-Tg MD4 B cells' survival in the spleens of unimmunized recipient mice. For experimental approach see **Fig. 3a**. **a**, Gating strategy for unproliferated (CD45.1$^{+}$ CFSE$^{high}$) and proliferated (CD45.1$^{+}$ CFSE$^{int/low}$) Ig-Tg and control (CD45.1$^{-}$ CFSE$^{high}$) B cells from the spleens of recipient mice. The examples shown correspond to 72 h post B cell transfer. The left and right panels are from B6 and TCRα$^{-/-}$ recipient mice, respectively. **b**, Ratios of unproliferated Ig-Tg B cells to control B cells in the spleens of recipient mice, normalized to the injected ratio of Ig-Tg to control B cells. Each symbol represents a single mouse. Data from n=5 independent experiments. **c**, **d**, Ig-Tg B cells' surface IgM$^{a}$ (BCR) (**c**) and fluorescent HEL binding (**d**) following *ex vivo* pulsing with the indicated doses of Ags and 3 h culture. Data from n=2 independent experiments, shown as mean ± SEM. **e**, Ratios of unproliferated Ig-Tg B cells to control B cells in the spleens of recipient mice 12 h after pulsing with the indicated Ags, normalized to the injected ratio of Ig-Tg to control B cells. Data shown as mean ± SEM. n=2 independent experiments with 4 mice. **f**, Representative plot of unproliferated and proliferated Ig-Tg and control B cells gated as in (**a**) from the spleens of recipient mice 72 h after sphDEL pulse and transfer. Representative of n=2 independent experiments with 4 mice.

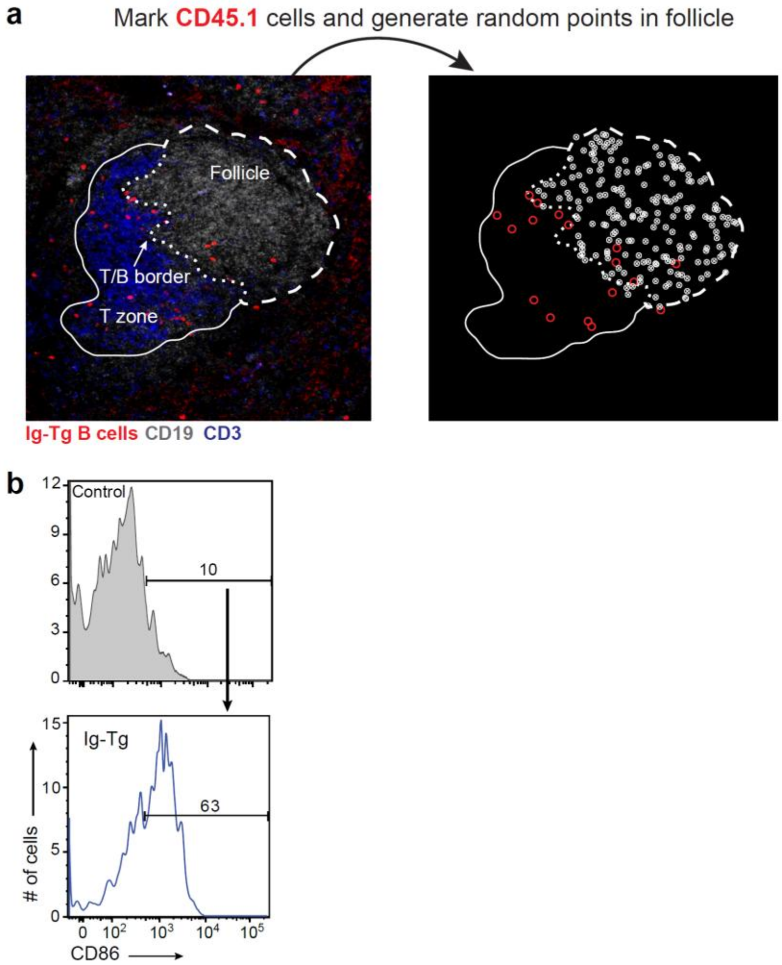


**Supplementary Figure 3 (relates to Fig. 4). Ag-primed B cells return to a quiescent phenotype in the absence of T cell help *in vivo*. a**, An illustration for quantitative analysis of Ag-pulsed Ig-Tg B cells' distance to the T/B border in spleen sections stained with CD19-, CD3-, and CD45.1-specific antibodies (see **Fig. 4a–c**). The follicle, T cell zone, and Ig-Tg B cells were marked in ImageJ and their coordinates read into a Matlab script which generated random points in the follicle and calculated the distance to the T/B border of each Ig-Tg B cell and randomly generated points. For each follicle the average distance to the T/B border of Ig-Tg B cells was then normalized to the average distance of randomly distributed points to derive normalized average distances in **Fig. 4c**. **b**, Determination of the "positive" gates for Ig-Tg B cells (See **Fig. 4 e**, **i**, **k**). The brightest 10% of control cells for a given marker were gated, and the gate applied to the Ig-Tg population; the fraction of the Ig-Tg population that fell within the gate was defined as positive.

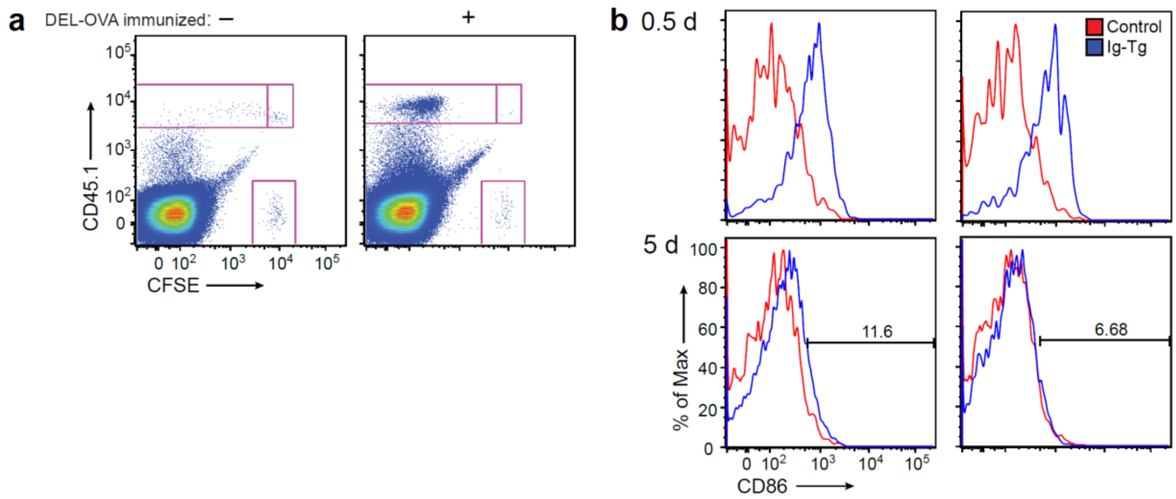


**Supplementary Figure 4 (relates to Fig. 6). Inactivated B cells can re-acquire Ag and participate in the T dependent humoral immune response. a**, Representative plots for unproliferated (CD45.1$^{+}$ CFSE$^{high}$) and proliferated (CD45.1$^{+}$ CFSE$^{int/low}$) Ig-Tg, and control (CD45.1$^{-}$ CFSE$^{high}$) B cells from spleens of non-reimmunized (left) and DEL-OVA reimmunized (right) secondary recipient mice 3 days post reimmunization. Plots representative of n=3 independent experiments with 5 mice per condition**. b**, Representative CD86 histographs of Ig-Tg and control B cells 0.5 d.p.t. (top) and 5 d.p.t. (bottom). Two histographs are shown for each timepoint. The fraction of Ig-Tg B cells expressing higher CD86 than control at 5 d was estimated as the difference in percentage of Ig-Tg and control cells that fell within the gate defining the brightest 5% of control cells. Plots representative of n=3 independent experiments with 6 mice.

| **Supplementary Table I. List of antibodies used** | | | | | | |
|---|---|---|---|---|---|---|
| **Target** | **Application** | **Species** | **Fluorochrome(s)** | **Clone** | **Dilution(s)** | **Manufacturer** |
| *B220* | FC | Rat | FITC, PerCP-Cy5.5, PE-Cy7, V500 | RA3-6B2 | 50, 100, 100, 50 | BD Pharmingen |
| *Bcl6* | IF | Mouse | Alexa 647 | K112-91 | 25 | BD Pharmingen |
| *CD3 without* | IF | Arm. Ham. | PE-Texas Red | 145-2C11 | 100 | BD Biosciences |
| *CD4* | FC | Rat | APC-Cy7 | RM4-5 | 100 | BioLegend |
| *CD4* | FC | Rat | V500 | RM4-5 | 400 | BD Biosciences |
| *CD8* | FC | Rat | APC-Cy7 | 53-6.7 | 100 | eBioscience |
| *CD8* | FC | Rat | V500 | 53-6.7 | 50 | BD Biosciences |
| *CD11c* | MACS | Arm. Ham. | Biotin | N418 | 100 | BioLegend |
| *CD19* | IF | Rat | Biotin | 6D5 | 100 | BioLegend |
| *CD19* | FC, IF | Rat | PE | 1D3 | 100 | BD Pharmingen |
| *CD38* | FC | Rat | PerCP-eFluor 710 | 90 | 200 | eBioscience |
| *CD43* | MACS | Rat | Biotin | S7 | 100 | BD Biosciences |
| *CD45.1* | IF, FC | Mouse | PE, Pacific Blue, Alexa 647, Alexa 700 | A20 | 50, 100, 100, 50 | BioLegend |
| *CD45.2* | FC | Mouse | Pacific Blue, Alexa 700, PerCP-Cy5.5 | 104 | 50, 50, 50 | BioLegend |
| *CD86* | FC | Rat | Biotin, Alexa 647 | GL-1 | 100, 100 | BioLegend |
| *CD95* | FC | Arm. Ham. | PE-Cy7 | Jo2 | 100 | BD Pharmingen |
| *CD138* | FC | Rat | biotin | 281-2 | 100 | BD Pharmingen |
| *CD197* | FC | Rat | biotin | 4B12 | 10 | BioLegend |
| *GL-7* | FC | Rat | Biotin, eFluor 450, eFluor 660 | GL-7 | 100, 100, 100 | eBioscience |
| *I-A/I-E* | FC | Rat | PerCP-Cy5.5 | M5/114.15.2 | 400 | BioLegend |
| *I-A$^b$/Ea* | FC | Mouse | Biotin | Y-ae | 100 | eBioscience |
| *IgD* | FC | Rat | PerCP-Cy5.5, APC-Cy7 | 11-26c.2a | 100 | BioLegend |
| *IgD* | IF | Rat | FITC | 11-26c.2a | 100 | BioLegend |
| *IgG$_1$* | FC | Rat | biotin | A85-1 | 100 | BD Pharmingen |
| *IgG$_1^a$* | ELISA | Mouse | biotin | 10.9 | 1000 | BD Pharmingen |
| *IgM$^a$* | FC | Mouse | PE | DS-1 | 200 | BD Pharmingen |
| *IgM$^a$* | ELISA | Mouse | biotin | DS-1 | 4000 | BD Pharmingen |
| *Vb5* | FC | Mouse | PE | MR9-4 | 200 | BD Pharmingen |
| *Streptavidin* | FC | n/a | Qdot 605 | n/a | 200 | Life Technologies |
| *Streptavidin* | IF | n/a | Dylight 488 | n/a | 100 | BioLegend |
| *Streptavidin* | IF | n/a | Alexa 647 | n/a | 100 | Life Technologies |
| **Legend** | | | | | | |
| FC – Flow Cytometry | | | | | | |
| IF – Immunofluorescence | | | | | | |
| MACS – AutoMACS purification | | | | | | |
